# Progress and challenges in decolonizing global health research partnerships: A scoping review

**DOI:** 10.1016/j.ghrp.2026.07.002

**Published:** 2026-07-31

**Authors:** Rebekah Kaufman, Mariana Garza Zuazua, Sarah Gallalee, Canice Christian, Lucía Abascal Miguel, Jane Fieldhouse, Elizabeth Fair, Ali Mirzazadeh

**Affiliations:** a Institute for Global Health Sciences, University of California San Francisco, San Francisco, CA, USA; b University of Texas at Austin, Austin, TX, USA; c Division of Pulmonary and Critical Care Medicine, University of California, San Francisco, CA, USA; d Columbia University Mailman School of Public Health, New York, NY, USA; e Department of Epidemiology and Biostatistics, University of California, San Francisco, CA, USA

**Keywords:** Decolonizing Global health, Research, Partnership

## Abstract

**Background:**

Despite recent calls for change, the field of global health continues to perpetuate unequal power dynamics due to colonization and disparate resource access. While there is research on the history of colonization in global health and a current push towards ‘decolonization,’ more research is needed to explore how global health partnerships between high-income countries (HICs) and low-middle-income countries (LMICs) can decolonize.

**Methods:**

We conducted a scoping review of peer-reviewed literature published between 2011 and 2024 focused on global health decolonization in collaborative partnerships between HICs and LMICs by searching Web of Science, PubMed, Embase, ProQuest databases, and JSTOR. Primary outcomes included descriptive information on models of decolonized global health in collaborative research partnerships, progress made in decolonizing collaborative research partnerships, barriers to decolonization in collaborative research partnerships, and roles of different contemporary stakeholders in the decolonization of collaborative research partnerships.

**Results:**

We included 56 studies in this review. Primary themes were identified, including: 1) Decolonized global health should be equitable, including fair and equal recognition of work and cultural humility should be centered; 2) Progress has been made in the decolonizing global health movement through increased acknowledgment of the topic in education and leadership; 3) Significant barriers persist, such as implicit bias, different standards, poverty imbalance, and a lack of authority held by persons from LMICs; and 4) Individual researchers, academic institutions, and structural level stakeholder can work to combat these barriers by shifting paradigms or current structures and methods.

**Conclusions:**

Findings from this review highlight the importance of equitable, participatory, and culturally humble approaches to global health research partnerships. These findings can inform future reform efforts by supporting the development of a practical checklist for decolonized global health practice to guide stakeholders in implementing these principles.

## Introduction

Global health, as defined by Koplan et al.,^1^ is a “study, research, and practice that places a priority on improving health and achieving equity in health for all people worldwide.” While the current widespread definition of global health centers on health equity, theorists have discussed the clear outgrowth of global health from practices of colonialization, colonial medicine, tropical medicine, and international health.^2^, ^3^, ^4^, ^5^ The practice of colonialism was often performed by high income countries (HICs).^4^, ^5^, ^6^

As many countries established colonies across Africa, Asia, India, and the Americas, new health challenges emerged through population displacement, labor systems, environmental changes, and the spread of infectious diseases associated with colonial expansion.^4^, ^5^, ^7^ Colonial systems also brought violence against those indigenous to the land through labor systems, spread of disease, and land capture, leading to increased medical needs and the spread of infectious diseases.^4^, ^5^, ^7^, ^8^, ^9^ Colonizing countries often focused on medical needs and diseases as they impacted their own ability to colonize land and conduct trade.^10^ Eventually, this led to the establishment of educational institutions such as the London School of Hygiene and Tropical Medicine (LSHTM) (1899), which studied endemic disease, thereby fundamentally establishing a connection between colonization and academia.^7^, ^9^, ^11^ Over time, tropical medicine and colonial medicine practices evolved into international health and, eventually, modern-day global health.^4^, ^5^

In the context of global health, decolonization generally refers to efforts to identify, challenge, and transform historical and contemporary power imbalances that privilege institutions, knowledge systems, and actors from formerly colonizing countries over those from previously colonized settings. Decolonization extends beyond increasing representation and seeks structural changes in leadership, funding, knowledge production, authorship, and decision-making processes within global health partnerships.^12^, ^13^ While most formerly colonized countries have achieved political independence, many scholars argue that colonial relationships persist through what Quijano described as the "coloniality of power." Under this framework, inequalities are reproduced through enduring systems of economic dependence, knowledge production, institutional authority, and social hierarchy that continue beyond formal colonial rule.^14^ In global health, these dynamics may manifest through unequal control of research funding, authorship, agenda-setting, leadership positions, and the privileging of Western epistemologies and knowledge systems.^15^, ^16^ Decoloniality refers to efforts to challenge and transform these enduring structures by promoting more equitable forms of partnership, representation, leadership, and knowledge production.^15^ Understanding decolonization through the lens of coloniality therefore provides a useful framework for examining contemporary global health collaborations.^14^, ^15^, ^16^

Despite changes, modern-day global health, and research collaborations within global health research, continue to perpetuate unequal power dynamics in partnerships between HICs and low-middle-income countries (LMICs).^2^, ^4^, ^5^ Even the terminology HIC and LMIC points to global health’s colonial history and a history of hierarchal systems.^17^ Notable inequities in global health research partnerships are seen across manuscript authorship, funding, and leadership.^18^, ^19^, ^20^ Authorship metrics have reported LMIC researchers are less likely to be first of last author on collaborative research.^18^, ^21^, ^22^ Research on funding distribution has also reported inequities such as the 10/90 gap; revealing only 10% of global health research funding concentrates on diseases that burden 90% of the global population, while leadership imbalances in global health organizations have also been measured reporting 83% of leaders in global health affiliated organizations are from HICs and 92% of leaders have degrees from HICs.^23^, ^24^

These concerns and inequities have led to the current decolonizing global health movement.^25^ Major global health institutions have released briefings addressing their colonial histories and how they plan to incorporate decolonization at their institution.^11^, ^26^ While there is vital research on the history of colonization in global health and a current push towards decolonization, actionable measures of decolonization in global health partnerships between HICs and LMICs are lacking.^27^ The primary goal of this study is to understand the extent of literature on the topic and to summarize existing literature on decolonization in global health research partnerships between HICs and LMICs.^28^

## Methods

### *Study design*

This scoping review was conducted to examine decolonization in global health research partnerships between high-income countries and low- and middle-income countries. The review methodology was developed and conducted in accordance with the Preferred Reporting Items for Systematic Reviews and Meta-Analyses Extension for Scoping Reviews (PRISMA-ScR) guidelines and the Arksey and O'Malley framework for scoping reviews.^29^, ^30^

### *Search strategy*

Literature searches were conducted in Web of Science, PubMed, Embase, ProQuest, and JSTOR. The original search was performed on January 3, 2021, and covered publications from January 2011 through 2021. To capture newly published literature, the same search strategy was repeated in the same databases on January 17, 2025, covering publications from 2021 through December 2024. Search terms included multiple spelling variations and combinations of keywords related to global health decolonization, including “global health,” “decolonization,” “hegemony,” and “supremacy.” The complete search strategies for all databases are provided in Appendix Item 2.

### *Inclusion and exclusion criteria*

Studies were eligible for inclusion if they met the following criteria: (1) published between January 2011 and December 2024; (2) published in a peer-reviewed journal; (3) discussed global health decolonization or related concepts, including supremacy and North-South partnerships, within the context of global health research partnerships between high-income countries and low- and middle-income countries; (4) included any peer-reviewed publication type, including original research, reviews, commentaries, editorials, and perspectives; and (5) were published in English.

Studies were excluded if they met any of the following criteria: (1) non-peer-reviewed literature, including books, dissertations, conference abstracts, reports, and other gray literature; (2) publications not focused on global health decolonization or related concepts; (3) publications not addressing partnerships or relationships involving both HICs and LMICs; or (4) non-English publications.

Detailed inclusion and exclusion criteria are provided in Appendix Item 1.

### *Study selection*

All records were imported into Covidence for screening. Duplicate records were removed prior to screening. Titles and abstracts were independently screened by multiple reviewers (JF, EF, SG, CC, JJL, LAM, AM, MGZ and RK) using the predefined eligibility criteria. Each abstract selected for inclusion was reviewed by at least two reviewers. Disagreements were resolved through discussion, with final adjudication by AM and EF when necessary. Full-text articles were subsequently reviewed by a single reviewer (RK, MGZ, SG, JF, EF, or AM) to determine final eligibility.

### *Data extraction*

Data were extracted and managed using Covidence and Microsoft Excel. Extracted information included article title, author names, journal, publication year, and relevant text related to the review objectives. Data were recorded using a standardized extraction form.

The review focused on four predefined domains of interest: (1) models of decolonized global health, (2) progress toward decolonization, (3) barriers to decolonization, and (4) the roles of contemporary stakeholders in decolonization. These domains guided data extraction and subsequent evidence synthesis.

### *Evidence synthesis and analysis*

For articles identified in the original search (2011–2021), thematic coding was conducted by a single reviewer using Preview. A codebook was developed and is provided in Appendix Item 3. Coding was conducted iteratively. Relevant quotations were extracted into Excel and assigned to one or more of the predefined domains of interest. Themes were identified from the extracted quotations and subsequently reviewed by two authors (RK and AM), who grouped related themes into overarching themes and subthemes within each domain. Representative quotations were selected to illustrate key findings.

For articles identified in the second search (2021–2024), GPT−4-turbo (OpenAI; accessed through ChatGPT Plus in March 2025) was used as an assistive tool to support thematic identification within the predefined domains of interest. Included papers were entered into GPT−4-turbo using a standardized prompt developed and refined by the authors. Prior to implementation, the prompt was tested against author-led reviews of sample manuscripts to evaluate the credibility and consistency of thematic identification. The final prompt was applied consistently across all included articles. The complete prompt is provided in Appendix Item 5.

GPT−4-turbo outputs were used to identify candidate themes within the predefined domains; however, final interpretation, refinement of themes and subthemes, selection of representative quotations, and synthesis of findings were performed by the authors. Supporting quotations were identified manually from the full-text articles.

Thematic saturation was assessed during the review process using guidance from Rahimi and Khatooni.^31^ GPT−4-turbo was used to assist in identifying whether newly included articles generated novel themes after groups of 10 and 25 articles were analyzed. Summaries of themes and subthemes generated at these intervals were reviewed by the authors and compared with themes identified from the original search. Final determinations regarding thematic and category saturation were made by the authors.

## Results

The original search of five electronic databases in January 2021 yielded 502 identified articles of which 31 met our inclusion criteria. The repeated search in January 2025 yielded 1758 articles, 60 were screened for full text inclusion, and 25 are reported in the results as determined by category saturation (Fig. 1).^30^, ^31^ The total search period covered was January 2011-December 2024. Overall, we included 56 studies in this review.

**Fig. 1:**
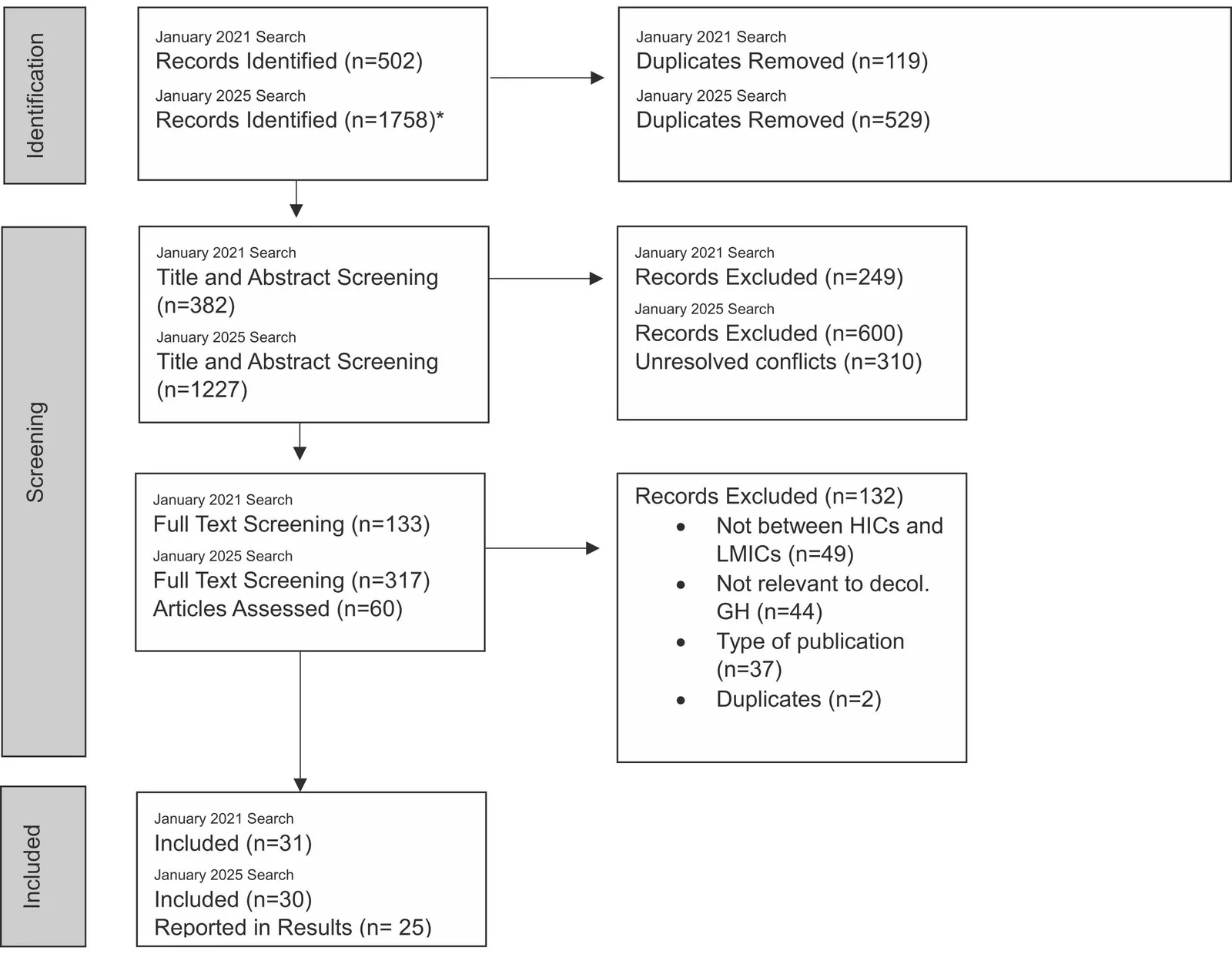
Flow diagram of search and selection strategy^29^, ^30^ *1758 articles were identified; however, two studies were manually identified as being included from the original search, making it 1756.

Findings were organized into four predefined domains of interest: (1) concepts and models of decolonized global health research partnerships, (2) progress toward decolonizing research collaborations, (3) barriers to implementing decolonized research partnerships, and (4) stakeholder roles in advancing decolonized research collaborations. Appendix Item 4 provides all articles affiliated with each theme.

### Concepts and models of decolonized global health research partnerships

Thirty-eight papers described potential models of decolonized global health research partnerships. Across studies, three recurring themes emerged: equity, participation and power sharing, and cultural humility and epistemic justice.

A. Equity

Equitable global health research collaboration means the removal of supremacy, reducing unfair power dynamics, equitable authorship, equal recognition, and the incorporation of a social justice and human dignity lenses into collaborative initiatives.^2^, ^32^, ^33^, ^34^, ^35^, ^36^, ^37^, ^38^, ^39^, ^40^, ^41^, ^42^, ^43^, ^44^ This would entail the removal of supremacy across countries, within academia, and the disassembling of colonial systems and white saviorism, with new systems being grounded in solidarity and liberation.^2^, ^35^, ^36^, ^37^, ^43^, ^45^ Structural change is required to reach equitable global health as colonial and neocolonial sentiments reinforce ideologies of hegemony.^2^, ^35^, ^36^, ^37^, ^38^, ^39^ More than just theoretical decolonization, decoloniality must exist at an ontological level.^40^ Specific examples of where inequitable structures exist in research collaborations are found in authorship, publication, conference presentations, research project tasks, and recognition.^21^, ^36^, ^42^, ^46^, ^47^ Decolonized global health would denote that LMIC researchers are meaningfully engaged and recognized as key authors when partnering with a HIC.^2^, ^21^, ^36^, ^37^, ^48^ Equitable global health means equal recognition of work, the value of all researchers globally, and co-leadership in research collaborations.^2^, ^21^, ^32^, ^33^, ^35^, ^36^, ^47^, ^49^, ^50^, ^51^

*“To decolonize global health is to remove all forms of supremacy within all spaces of global health practice, within countries, between countries, and at the global level. Supremacy is not restricted to White supremacy or male domination.”*^36^*(Abimbola S, pg 1627)*

*“Decolonization” in this context reaches beyond removal of colonial power and dismantling of colonial structures to include decolonization of the mind that made the colonizer feel superior and the colonized inferior by enforcing structural drivers of discrimination and barriers to self-determination.”*^2^*(Eichbaum QG, pg 329)*

*“To reimagine global health and shift power to people and institutions on the margins and the periphery, everyone in global health must advocate for and activate two forces: leadership by the Global South and allyship by the Global North. It is the synergy of these dual forces that is required to disrupt the status quo and tip the scales in transforming global health into a model rooted in justice, equity, global solidarity, and collective liberation.”*^*43*^*(Pai, pg 1713)*

Across the literature, equity was commonly linked to structural change, suggesting that individual-level reforms alone are insufficient without addressing broader systems that reproduce colonial and neo-colonial power relations.

B. Participation and Power Sharing

‘Participatory models emphasized local leadership, Indigenous representation, reciprocity, inclusion, community engagement, and shared decision-making.^2^, ^33^, ^36^, ^39^, ^41^, ^44^, ^45^, ^47^, ^50^, ^51^, ^52^, ^53^, ^54^, ^55^, ^56^ Global health leadership should come from those who are marginalized by coloniality.^2^, ^36^, ^39^, ^54^ Decolonized global health research collaboration centers solidarity rather than charity and research collaborations between HICs and LMICs are not unidirectional, emphasizing co-ownership, co-construction, and shared decision making throughout the entire research continuum.^2^, ^12^, ^32^, ^33^, ^34^, ^36^, ^44^, ^50^, ^51^, ^52^, ^57^, ^58^, ^59^ Reciprocity should exist between partnerships where the flow of knowledge is not unilateral and travel is reciprocal between countries, not exclusively HIC researchers traveling to LMIC settings.^2^, ^42^, ^43^, ^44^, ^53^ Lastly, the focus of research in HIC and LMIC partnerships should be “with” each other, rather than research “on” the LMIC counterpart.^60^

*“In this imagined future, global health practitioners in HICs and those who are otherwise privileged, have embraced an appropriately modest view of their importance, and mastered the art of critical allyship, where they see their primary role as allies and enablers rather than leaders. Rather than drawing from a limited talent pool of elite HIC institutions, Black, Indigenous, and other people of colour are the real leaders of global health. In particular, women in the Global South, who form the majority of the global health workforce, are proportionately represented in leadership.”*^36^*(Abimbola S, pg 1628)*

*"Where bidirectional exchanges exist, it is well known that HIC learners traveling to LMICs consistently outnumber LMIC learners traveling to HICs. One explanation for this imbalance is that HIC institutions assume their students are always “helping” in under-resourced settings. Yet, on the contrary, visiting HIC students often place a burden”*^2^*(Eichbaum QG, pg 334)*

*“The inclusion dimension reflects the ethical significance of self-determination. It calls for meaningful participation in decision-making processes by all those affected, particularly those affected who are considered socially marginalized or disadvantaged. The dimensions can each bear on one another. For instance, having an unequal playing field will clearly mean that some people are less well situated to achieve human flourishing.”*^*33*^*(Pratt B, pg 627)*

Rather than positioning LMIC partners as beneficiaries of research, studies consistently advocated for co-ownership and co-construction of research agendas, implementation, and dissemination activities.

C. Cultural Humility and Epistemic Justice

Global health collaborations should promote cultural humility, listening, reflecting, learning from others, and remove itself from western, or Eurocentric epistemologies.^13^, ^39^, ^40^, ^44^, ^45^, ^46^, ^51^, ^56^, ^59^, ^60^, ^61^, ^62^, ^63^

Human rights should be at the core of global health practice.^2^ In addition global health research should center around community-oriented practice and pluriversality in education, knowledge, and practice.^39^, ^42^, ^60^ Pluriversality, in contrast to western universalism, incorporates different knowledge practices and ways of being.^40^, ^59^ Western universalism on the other hand is connected to “western supremacy” and assumes western frameworks are inherently the best and can be applied to all populations.^2^, ^39^, ^42^, ^60^, ^63^, ^64^ However, westerners do not represent the global majority.^40^, ^63^ Basing and applying frameworks for global health practice with non-western populations from research or knowledge informed by western populations prevents respect, humility, and decoloniality.^39^, ^48^, ^60^, ^63^ Understanding different ways of conceptualizing health allows movement away from coloniality towards decolonized thought, education, and research.^42^, ^65^

*"A decolonising research methodology is an approach that is used to challenge the Eurocentric research methods that undermine the local knowledge and experiences of the marginalised population groups."*^60^*(Keikelame MJ, pg 1)*

*"The Global North Cartesian tradition in which Being is divided into body and mind and spirit is ignored, disregards most non- Global South traditions, including those of Indigenous peoples of North America, in which mind, body and soul/spirit are inextricably linked not only to each other within one person but to other people, nature, the environment and collective notions of Spirit. The space of configuration of disease encompasses all of these entities collectively and interchangeably."*^48^*(Redman-MacLaren M, pg 744)*

Several studies distinguished between colonialism and coloniality. While colonialism refers to historical systems of political domination, coloniality refers to the enduring power structures, hierarchies, and epistemologies that persist after formal colonial rule. Decolonial approaches therefore seek not only institutional reform but also transformation of the assumptions and knowledge systems that continue to shape global health practice. Studies frequently critiqued Western universalism and advocated for pluralistic approaches to knowledge generation and application.

### Progress toward decolonizing research collaborations

Twenty-two papers reported evidence of progress toward decolonization. Three broad areas of progress were identified: increasing awareness, changes in leadership and representation, and the development of initiatives focused on equity and capacity strengthening.

A. Progress in Awareness

Increased education, social movements, and advocacy on decolonization represent progress in knowledge and discourse that can be applied to research partnerships.^34^, ^50^ Student-led social movements at academic institutions have pushed decolonization to be centered on institutions and curriculums.^35^, ^38^, ^42^, ^66^ Additionally, education on the social determinants of health, decolonial theory and praxis, health equity, and human dignity in global health curriculums marks progress made.^34^, ^38^, ^42^, ^52^, ^66^ The sharing of narratives using social media has increased awareness; for example, the Saga Santé Project Facebook page has provided a space for African youth to share their experiences and views on health issues by which they are directly impacted.^64^ Providing opportunities to openly share narratives allows local and individual voices to be represented.^64^ Increased awareness and education on the topic of decolonizing global health facilitates more movement to be made in the field across academic institutions.^33^, ^35^, ^38^, ^42^, ^66^

*"Importantly, these movements are often student-led and include high proportions of students from LMICs and their diasporas. Where we work, at the London School of Hygiene and Tropical Medicine, the Decolonizing Global Health (DGH)movement has started exploring concrete ways in which global health teaching and research can overcome its colonial past in terms of representation, research and funding processes. These movements, which have emerged predominantly within universities in the Global North, are only one of many manifestations that critiques of our current global health system have taken."*^66^*(Lawrence DS, pg 519)*

Across studies, awareness-building initiatives were frequently student-led and were associated with efforts to revise curricula, increase discussion of colonial legacies, and promote health equity frameworks within academic institutions.

B. Progress in Leadership

Progress in leadership can be seen through multilateralism and the WHO Regional Offices potentially impacting research collaborations.^67^, ^68^, ^69^ Despite the development of the WHO and its regional offices being disproportionately Eurocentric and western-led, the development of regional offices did allow for greater representation and leadership.^69^ However, power dynamics within regions still exist, as notably, the last WHO regional office to be formed was Africa.^68^, ^69^ A shift towards projects and partnerships focused on building local capacity strengthens local representation and leadership.^21^, ^48^, ^57^, ^67^ Lastly, academic journals have increased diversity and LMIC leadership among editors and editorial boards as well as waived publication fees for authors from LMIC institutions.^12^

*"Although global health still grapples with legacies of paternalistic philanthropy, colonial power asymmetries and ways of thinking, and/or affirmations of national self- interest, multilateralism as a political stance reaffirms its mission and provides direction to its practice."*^68^*(Agartan TI, pg 201)*

Several studies described increased representation of LMIC scholars within editorial boards, academic leadership positions, and international organizations. However, studies also noted that gains in representation have not always translated into substantive shifts in decision-making authority, suggesting ongoing tensions between symbolic inclusion and structural change.

C. Progress in Paradigm, Initiatives, and Capacity Building

Academic institutions and global health organizations have begun to address colonial legacies still existing in research collaborations.^49^, ^66^ For example, schools such as LSHTM, formed a group to discuss the institution’s colonial connections and ways in which they can reconstruct systems and trust.^65^ Additionally, academic institutions have explored methods such as representation to correct past injustices.^65^ Institutions addressing legacies is a vital first step in the right direction.^49^, ^66^ Bidirectional exchange and capacity building initiatives have also been explored in recent years to emphasis equity in resources, training, leadership, authorship and education as applied to research collaborations between HICs and LMICs.^47^, ^50^, ^53^, ^54^, ^70^ Initiatives have also been undertaken by US National Institutes of Health (NIH) to directly fund and support researchers at foreign intuitions.^12^

*“With respect to Access and Representation, we strongly endorse bidirectional knowledge transfer, educational and travel opportunities, freely accessible medical education, and research, emphasizing the expertise and perspectives of Emergency Medicine (EM) leaders in LMICs. Some recent examples demonstrating positive changes in the Global Emergency Medicine (GEM) field include resources from the African Federation of Emergency Medicine (AFEM). AFEM provides comprehensive EM educational and clinical resources that are open access, free, and implemented worldwide. An additional two examples are found within the University of California San Francisco (UCSF) specialty-independent Health, Equity, Action, and Leadership (HEAL) fellowship, which aims to develop bidirectional education, interprofessional exchange, and partnership-based sustainable work over multi-year assignments. Simultaneously, UCSF has created a Center for Health Equity in Surgery and Anesthesia, which focuses on local and global health equity featuring bidirectional research, advocacy, education, and training. These frameworks, put into practice by AFEM and UCSF, can be easily molded to other global health initiatives.”*^*70*^*(Dozois, pg 7)*

*“In recent decades, the global community has amplified efforts to bridge this Global North (GN) - Global South (GS) disparity, including by increasing health research funding and adopting new funding models to GS institutions and countries, supporting research education, implementing health research capacity-strengthening programmes, and supporting partnerships, networks and collaborations. New networks and GS-led initiatives to address GN–GS disparities are also emerging, such as the Global Health Decolonization Movement in Africa. Many such initiatives have had positive effects, increasing capacity and enabling some GS institutions and countries to improve research performance and attain a pioneering research position compared with their neighboring countries.”*^*47*^*(Abouzeid M, pg 3)*

*“The jolt has shaken up the system, and these fault lines are being repaired by increased sensitization to these issues and necessary rectification. This includes better focus on development of local Human Resources, increase in proportion, and diversity of local trained staff across every domain from healthcare to logistics to administration. Additionally, several explicit guidelines and constructive strategies, including what to do and what not to do — for the journals and journal editors, collaborating research institutions and funders — have been developed to ensure a truly equitable non-hierarchical partnership, including authorship credits, in North-South GH&GS collaborations”*^50^*(Chawla, pgs 259–260)*

Studies reported growth in bidirectional exchange programs, locally led research initiatives, and direct funding mechanisms designed to strengthen research capacity. Collectively, these initiatives suggest movement toward more equitable partnerships, although the literature generally viewed these efforts as early steps rather than evidence of complete transformation.

### Barriers to implementing decolonized research partnerships

Thirty-six papers discussed barriers to decolonization. Four recurring themes emerged: implicit bias and knowledge exclusion, unequal standards and practices, financial inequities, and power imbalances.

A. Implicit Bias, Undervaluing, and Knowledge Exclusion

Implicit biases encompassing racism, paternalism, saviorism, Eurocentric epistemologies and othering are significant barriers and individual researchers, institutions, and governments must self-reflect to properly engage in global health practice and partnership.^35^, ^38^, ^49^, ^56^, ^64^, ^67^, ^71^

Implicit biases and the dominance of Western institutions and epistemologies can contribute to the undervaluing of LMIC researchers, exclusion of local knowledge, and unequal participation in research collaborations and exclusion of local community voice.^35^, ^38^, ^47^, ^49^, ^51^, ^53^, ^64^, ^67^, ^70^, ^71^, ^72^ Additionally, western universalism, and the exclusive use of biomedical approaches represent a significant barrier to decolonized global health practice.^35^, ^38^, ^49^, ^64^, ^67^, ^71^ The pervasive nature of implicit biases, often structured by colonial attitudes, makes overcoming racial biases vital to global health.^36^, ^38^, ^49^, ^67^ Western practices often focus on hyper-individualized care, biomedical practices, and cartesian knowledge.^40^, ^63^, ^71^ Applying these frameworks to non-western populations may not only be ineffective but does not reflect respect or cultural humility in collaborative research.^40^, ^62^, ^63^, ^71^ Language, or the dominant use of English in academic publishing, conferences, and grants also acts as a barrier, exemplified by the fact that individuals in decision making positions often come from predominantly English-speaking countries, resulting in exclusion, while biased visa policies also impact participation in conferences and forums.^12^, ^43^, ^59^, ^70^

*“This has given rise to over-inclusive and arbitrary descriptive terms in Global North cultures, such as the “Third World,” and the “Orient.” In essence, the previous role-play of the colonial power and the colonized nations continues far beyond formal colonialism, through sustained international influence and attitude.”*^49^*(Kim JU, pg 403)*

*"International coverage of the continent, however, instead has focused on the assumed inevitable failure of African nations to effectively respond to the pandemic, failures which are often caused by limited resources resulting from colonialism and modern-day imperialism. This representation is obviously biased, and is so because those with power to control the narrative around the pandemic continue to be disproportionately not from or based in the ‘Global South’.”*^35^*(Büyüm AM, pg 3)*

*“English language supremacy is rooted in colonial and Eurocentric theories of knowledge. The adoption of British and American English as standard language in much of global health furthers this, reproducing Anglophone hierarchies of linguistic respectability in global health. This also presents barriers to the dissemination of global health research, with translation into local language often required to communicate findings for patient populations and wider audiences in settings in which the research was conducted, thus prioritizing the “foreign gaze” over the “local gaze”*^*12*^*(Ngaruiya, pg 9)*

*“Many trainees from HICs who visit LMICs expect to serve in a teaching role, either due to institutional suggestions or because of their own internal biases regarding the superiority of the education granted to them. Alternatively, scholars from LMICs, from trainees to well-established senior faculty, are much less frequently asked to serve as educators in HICs, even when they may hold the greatest experience. This common unidirectional model of HIC trainees traveling to LMIC sites perpetuates the colonial mentality present in GEM by viewing those from LMIC’s as less capable or knowledgeable than those trained in HICs.”*^70^*(Dozois, pg 4)*

B. Different Standards

Studies identified differences in ethical standards, research practices, and accountability mechanisms as persistent barriers.^2^, ^36^, ^49^, ^64^, ^65^, ^66^, ^73^, ^74^ The lack of universal ethical guidelines means researchers from HICs are held to different standards when practicing in their own countries than in LMICs.^2^, ^36^, ^49^, ^64^, ^65^, ^66^ This barrier has often led to lower clinical standards and irregulated rules perpetuating colonial legacies of medical experimentation, such as seen in the zidovudine trials, where in Asia and sub-Saharan Africa, a no-treatment control group was used.^2^, ^36^, ^49^, ^64^, ^65^, ^66^ Additionally, in March of 2020 two French scientists made remarks suggesting using Africa as a place to test covid vaccines, which led to push back from the international community that this suggestion represented a colonial mentality.^35^, ^37^ HIC’s approach to global health also often involves parachute or helicopter research and underqualified practitioners working in LMIC settings.^36^, ^45^, ^47^, ^49^, ^58^, ^65^, ^66^ Such approaches do not facilitate long-term sustainable partnerships.^2^, ^36^, ^45^, ^47^, ^49^, ^64^, ^65^, ^66^

*"Global North’s willingness to lower the clinical standard in the Global South. Turning the statement around, they revealed the powerless- ness of the countries of the Global South, and the subsequent domination by the Global North, much in parallel with other forms of neocolonial exploitation. What informed these decisions – economic or scientific reasons?"*^49^*(Kim JU, pg 403)*

*"Additionally, the short-term, changing rotations of students, money, and supplies can create a cycle of dependence on foreign medical schools for clinics and hospitals in the Global South. By not acknowledging root causes of a punishingly unfair and uneven distribution of wealth and the continued disenfranchisement of racialized people, especially women all over the globe, Northern medical school’s involvement will tend to reinforce power relations rather than disrupt them. This is analogous to treating the symptoms rather than the causes of illness and unintentionally making the situation worse."*^65^*(Bhandal T, pg 40)*

Several authors interpreted these disparities through the lens of coloniality, arguing that unequal standards reflect enduring assumptions about whose lives, expertise, and institutions are valued within global health systems.

C. Financial and Resource Imbalances

Funding structures were frequently described as reinforcing dependence and limiting local leadership.^41^, ^47^, ^66^, ^73^, ^75^, ^76^, ^77^ Funding for research projects disproportionately allocates resources to research for diseases that impact HICs.^41^, ^66^, ^73^, ^75^, ^76^, ^77^ Further, in some LMICs research is underemphasized relative to teaching and it may require the use of personal funds, while access to mentorship resources, effected by “brain drain” and technical resources also may differ, leading to imbalances ^73^ External debt repayments also further global poverty imbalances for LMICs to HICs, which prevent countries from spending on public goods that may lead to positive health externalities.^75^ The grant funding system also reflects inherently neocolonial ideals through indirect disbursement of funds, preventing local leadership initiatives.^41^, ^43^, ^47^, ^53^, ^55^, ^66^, ^75^, ^76^, ^77^ Rather than grant funds going directly to academic institutions in LMICs, they flow through a HIC institution which then has the agency to disburse funding, creating funding imbalances in research collaborations, while limiting collaborations between LMICs due to HIC collaborator requirements.^34^, ^41^, ^43^, ^47^

*"The existing Development Assistance for Health (DAH) system grew out of the post–World War II period of reconstruction and decolonization in which neoliberal principles of national interests, charity, or enlightened self-interest guided foreign aid from donors to recipients within a hierarchical and asymmetric relationship. Empirical evidence on donor priorities suggests that these motivations persist today"*^77^*(Ruger JP, pg 23)*

*"45 countries spent more on external debt payments than on health care in 2019, with percentage of government revenue spent on debt repayment exceeding health-care expenditure by 20–36% in 8 countries"*^75^*(Khan M, pg 897)*

*"From the way that much UK funding systems work, most grants are typically awarded to, and managed by, a designated lead institution, predominantly a UK university, which in turn disburses funds to their partners."*^41^*(Bucher A, pg 682)*

*“Universities in LMICs serve as educational bodies with greater emphasis on teaching than research. Protected research time is rare, and research-based promotion opportunities are ambiguous and limited. Investigators are expected to use personal funds for research activities, such as conferences; however, financial compensation for research is poor. Many investigators must support themselves through heavy teaching loads or secondary employment in private sectors, thus limiting their time for research. We, the Ghanaian authors, are among the many LMIC investigators who conduct our research on our own time during nights and weekends.”*^73^*(Anane-Binfoh N.A., pg 2)*

Across studies, financial inequities were consistently linked to broader power asymmetries, particularly when funding decisions, resource allocation, and accountability mechanisms remained concentrated within institutions in HIC settings.

D. Power and Leadership Imbalances

Authorship imbalances, lack of representation in leadership positions, lack of capacity building specific funding, inauthentic capacity building initiatives, geopolitical power dynamics, and the effects of COVID−19 all preserve HIC's agency and authority in research partnerships.^12^, ^21^, ^22^, ^34^, ^47^, ^49^, ^54^, ^55^, ^58^, ^75^, ^76^ Overall, LMIC authors are significantly underrepresented in the authorship of publications, and they notably lack recognition in vital positions such as first or last author and academic journals often bias towards Eurocentric epistemes.^12^, ^21^, ^22^, ^35^, ^36^, ^47^, ^56^, ^76^ Partnerships between HICs and LMICs have inherently hierarchical relationships, due to historical legacies of colonialism, with leadership often coming from HIC researchers and circumstances where LMIC researchers are not included in project conception.^36^, ^47^, ^54^, ^55^, ^58^, ^59^, ^76^

"*LMIC authors are profoundly under- represented, representing less than 50% of first authors and in some cases, less than 50% of all authors. Some of these papers have shown that LMIC author representation decreases in the prime first or last positions compared with overall representation."*^21^*(Hedt-Gauthier BL, pg 2)*

*"Global health institutions based in the ‘Global North’, often lacking representation of key communities at the decision-making table, end up perpetuating a Eurocentric worldview that does not adequately consider most of the world’s needs."*^35^*(Büyüm AM, pg 3)*

*“Despite recognition of the importance of GS health research leadership, the reality remains that power imbalances and inequities continue to exist in research and in international collaborations, with GN countries, institutions and researchers often acknowledged as the research “leaders”, including through being assigned research leadership roles of first or corresponding author on collaborative publications”*^47^*(Abouzeid M, pg 3)*

Notably, these findings were among the most consistently reported barriers across the reviewed literature, suggesting broad agreement that decision-making power remains unevenly distributed within global health partnerships.

### Stakeholder roles in advancing decolonized research collaborations

Thirty-six papers discussed stakeholder roles in advancing decolonization. Themes clustered around actions by individual researchers, academic institutions, and structural-level actors including governments, funders, journals, and international organizations.

A. Individual Researchers

The role of individual researchers in decolonizing global health research partnerships includes learning about the history of colonialism and white supremacy, as well as their current structural presence in global health.^2^, ^36^, ^40^, ^45^, ^62^, ^78^ In addition, self-reflection and relearning knowledge that perpetuates western universalism and implicit biases at an individual level is required.^2^, ^36^, ^40^, ^45^, ^52^, ^53^, ^62^, ^78^ Individual researchers from HICs can innately reflect colonial legacies if they do not shift their perspective to reflect “critical allyship” as their role in global health research supported by ensuring equitable recognition, shared research priorities, immersion within communities, and clinical equipoise.^2^, ^36^, ^44^, ^51^, ^59^, ^79^ Individual researchers should also engage in research that centers and uplifts the voices of the local community through ethnographic research methods to ensure local and community narratives are reflected.^2^, ^36^, ^40^, ^62^, ^78^ Additionally, focusing on locally led community-based research can prevent colonial legacies from being perpetrated by individual researchers today.^2^, ^35^, ^40^, ^53^

*"Preparing to work from a culturally humble, decolonizing framework requires learning about the historical, political, economic, and structural legacies of colonialism and White supremacy. Visitors can seek counter-narratives to dominant myths about the “Other,” and discuss strategies for de-centering Western hegemony in favor of acknowledging the legitimacy of indigenous and previously subjugated knowledge and experiences, particularly in relation to ways of understanding health, illness, and healing. Humility, openness to the unfamiliar, and commitment to learning create a basis for developing knowledge of the host community."*^62^*(Potash JS, pg 76)*

*“In this imagined future, global health practitioners in HICs and those who are otherwise privileged, have embraced an appropriately modest view of their importance, and mastered the art of critical allyship, where they see their primary role as allies and enablers”*^36^*(Abimbola, pg 1628)*

*"This must necessarily begin with a deconstruction and dismantling of the concept of humanity as currently conceived, the uncoupling of Man from human and the unsettling of the hegemonic western universalist conception of the human which normalises and over-represents the (western bourgeois) Man.”*^40^*(Affun-Adegbulu C, pg 2)*

Collectively, these studies suggest that individual researchers are expected to act not only as knowledge producers but also as reflexive partners who actively challenge unequal power dynamics within collaborations.

B. Academic Institutions

HIC academic institutions need to shift education and partnership paradigms to expand beyond competency-based education and western scientific epistemologies to better reflect pluriversality in knowledge and ethics.^42^, ^46^, ^57^, ^66^, ^74^ Education programs should teach the connection of global health to colonialism in order to augment awareness and should implement learner milestones centering decolonial global health praxis, bias training, navigating ethical dilemmas, and equitable collaboration.^12^, ^33^, ^34^, ^45^, ^51^, ^52^ Beyond education, diverse leadership at academic institutions can create representative global health education and research; while building sustainable and equitable partnerships rooted in humility and shared decision making as well as encouraging capacity building initiatives can mitigate structural inequities.^42^, ^43^, ^45^, ^51^, ^53^, ^54^, ^59^, ^60^, ^63^, ^65^, ^66^, ^80^ Relationships between institutions can use a memorandum of understanding (MOUs) to ensure partnerships recognize equal roles of researchers and institutions within partnerships.^60^ Academic institutions can also incorporate community perspectives in institutional review boards (IRBs) to mitigate differences in study perceptions between an institutional review boards (IRB) board and the community.^74^

*“This is increasingly relevant, as the trend seems to be for institutions in HICs to increasingly build links with those in LMICs. International researchers are a heterogeneous group, some of whom are respectful of institutional culture and reflective on their position within it, whereas others are less so. We all need to be culturally and racially literate when it comes to global health research and this needs to happen as early in our careers as possible. Medical schools and research institutions have a duty to generate discussion concerning the complexity of global health research and to develop, in partnership with their collaborators, guidelines for the responsible conduct of research in LMICs"*^66^*(Lawrence DS, pg 521)*

*“In addition, the design of MOU’s can be enabled by using strategies such as mediation and setting up formal partnerships that recognises the role of indigenous people from being ‘researched’ to ‘researchers”*^60^*(Keikelame MJ, pg 3)*

Across studies, institutional accountability was repeatedly emphasized, suggesting that meaningful decolonization requires changes to educational curricula, governance structures, partnership agreements, and research oversight processes rather than reliance on individual goodwill alone.

C. Structural Level Stakeholders: Governments, Global Health Organizations, Academic Journals, and Funders

Shifting to Shared Health Governance (SHG), or policy that reflects global justice theory through mutual accountability and representation of local players, can help break cyclical oppression.^36^, ^71^, ^77^ For SHG to exist, implicit hierarchical functions must be disbanded.^36^, ^71^, ^77^ Hierarchy, in global health, is reflected when global health organizations’ headquarters are prioritized over local or regional offices and when leadership exclusively comes from HIC institutions; collaborative leadership can encourage community engagement and redistribute power.^36^, ^54^, ^71^, ^77^ Leadership should draw from those who are marginalized by neocolonial systems.^36^, ^71^, ^77^ For example, indigenous and Black individuals, particularly women and those from LMIC institutions, should be prioritized as global health leaders.^36^, ^71^, ^77^ Direct and diversified funding of LMIC researchers focused on local priorities and funding specific to capacity building can also be used to overcome structural inequities and augment research infrastructure in collaboration with emphasis on building research infrastructure at a government level.^47^, ^53^, ^55^, ^58^, ^70^, ^80^ Governments and governmental organizations can also discourage hoarding of medical technology, such as vaccines, reform their visa policies, offer debt relief, and promote anti-racism initiatives ^43^ Grant applications and journals can encourage reflexivity, reduce processing fees, provide authorship reporting recommendations, and support diversity in study leadership, while governing bodies such as the World Health Organization can encourage the application of these techniques.^12^, ^22^, ^34^, ^43^, ^44^, ^56^, ^70^

*“Global health policy must reflect (a) a consensus among global, national, and subnational actors on common goals and measurable outcomes; (b) mutual collective accountability (for example, for resource use, implementation, and results); and (c) health agency–enhancing processes. Cost management and efficiency are integral parts of SHG.”*^77^*(Ruger JP, pg 25)*

*“It is a supremacist way of seeing and doing when we entertain implicit hierarchical assumptions - for example, about the headquarters of a global health organization being more important than its regional or country offices. Supremacy manifests in seeing the big as superior to the small - for example, in the focus on national governments when subnational governments are more consequential and closer to the ground”*^36^*(Abimbola S, pg 1627)*

Taken together, these findings indicate that decolonization is largely viewed as a redistribution of power, resources, and decision-making authority. While individual and institutional actions were considered important, most studies argued that lasting change ultimately depends on reforms to the broader systems that govern global health funding, leadership, publication, and accountability.

## Discussion

The assessed literature demonstrates a vision of decolonized global health that is equitable, participatory, and grounded in cultural humility. Viewed through the framework of coloniality, these findings suggest that many contemporary inequities in global health partnerships are not simply the result of differences in economic resources between HICs and LMICs, but rather reflect enduring systems of power, knowledge production, and institutional authority that persist beyond formal colonial rule. While HIC and LMIC classifications were used in this review to define study eligibility, many included studies described these dynamics using the concepts of the Global North and Global South to emphasize historical and political relationships that extend beyond economic categorization.

Notable findings in our review included that major barriers to equitable global health are implicit biases, different standards, financial imbalances, and lacking LMIC authority in global health.^21^, ^35^, ^36^, ^38^, ^49^, ^77^ Overcoming these barriers is a crucial role of contemporary stakeholders.^2^, ^36^, ^40^, ^66^ Individual researchers from HICs are responsible for understanding and unlearning implicit biases, hierarchy, western universalism, and learning cultural humility for equitable partnerships to exist.^2^, ^36^, ^40^, ^78^ HIC institutions need to ensure education systems expand beyond competency-based education and emphasize cultural humility.^42^, ^57^, ^66^ More so, academic hierarchy should shift away from funding and leadership predominately coming from HICs.^2^, ^36^ Institutions participating in parachute research should consider if this is a sustainable collaboration or if the main benefit is to their institution.^2^, ^36^, ^49^, ^64^, ^65^, ^66^ While knowledge and education on decolonization have significantly increased, greater LMIC leadership representation is needed in discussions about global health research partnerships.^35^, ^36^, ^66^, ^81^ A hierarchy in academia between HIC and LMIC institutions has no place in global health partnerships and equity, respect, and reciprocity should be at the core of global health collaborations.^2^, ^36^, ^49^ While there are many barriers to decolonizing global health, solidarity across LMIC and HIC individuals and institutions is a vital next step in moving towards a form of global health that we can call equitable.^36^ Novel definitions of global health, such as published by Jacobsen et al., have incorporated the importance of diverse perspectives within their frameworks potentially capturing the fields transition towards frameworks reflected in this scoping review.

Structural concerns at governing and funding levels are also essential to address for systemic change towards equity in global health collaborations.^34^, ^43^, ^47^ Within this review, reports of inequity in grant disbursement, leadership, and governance represent structural factors where alterations are needed.^34^, ^36^, ^43^, ^44^, ^47^, ^58^ In application this may include administrators at a government level and in organizations such as the National Institute of Health, the World Bank and Global Fund, and the Bill and Melinda Gates Foundation, who make decisions on how funding is allocated, modifying systems so money does not predominately flow through HICs.^19^, ^41^, ^66^, ^75^, ^76^, ^77^, ^82^ Notably, 88% of Bill and Melinda Gates Foundation grants are led by HIC institutions, 70% of Fogarty grants support HICs, and 73% of Welcome Trust grants are UK-based.^19^ This structure enables existing power dynamics through forced paternalism, limiting research focusing on local needs and true bidirectional collaboration.^21^, ^23^, ^35^, ^36^, ^43^, ^75^, ^83^, ^84^ For example, diseases such as tuberculosis, Chagas disease, and malaria have been historically underfunded and neglected despite their burden in LMICs.^23^, ^83^ Beyond individual project funding, intellectual property agreements and medical technology hoarding cause inequities such as vaccine apartheid.^43^, ^85^, ^86^ Increased manufacturing capacity in LMICs and intellectual property waivers have been explored in reference to COVID−19 vaccination development inequities, reflecting progress, however these initiatives can still emulate global power imbalances.^87^

As a field, global health should prioritize transitioning from charity-based frameworks to mutual aid frameworks.^88^, ^89^ Centering around grassroots organizations and local community organizations can increase reciprocity and local leadership in global health research collaborations.^2^, ^35^, ^40^, ^90^ Overall, mutual aid encourages local and participatory engagement and may help in the transition away from unilateral global health practices such as parachute research.^49^, ^66^, ^88^, ^89^ Emphasis on community-based practices and cultural humility in global health education and training can shift how global health researchers engage in collaboration research.^12^, ^33^, ^34^, ^45^, ^52^, ^53^ Global health education and training modifications that emphasize these practices and augment diverse perspectives can lead the shift in global health research practice and partnership.^12^, ^33^, ^34^, ^45^, ^52^, ^53^ Global health education programs may also consider emphasizing structural determinants and critical theory to contextualize global health inequities.^12^, ^33^, ^34^, ^45^, ^52^ Ratner et al. discuss global health education milestones mirrored to the socioecological model, where structures and systems, the outer ring of the model, are reflected at the highest learner level, when trainees see power dynamics and structural advocacy at a systems level.^52^

Cultural humility emerged as one of the few concepts that appeared across all four domains of the review.^39^, ^48^, ^60^, ^63^ It was reflected in proposed models of equitable partnership, identified as a mechanism for addressing implicit biases, incorporated into recommendations for education and training, and frequently discussed as a characteristic of effective leadership and collaboration. This consistency suggests that cultural humility may serve as an operational pathway through which broader goals of decolonization can be translated into everyday research practices and institutional relationships.

To address best practices at multiple levels global health researchers may consider exploring standardized frameworks which can outline processes that result in equitable global health research collaborations. Checklists that outline best practices can amalgamate recommendations developed through discussions with diverse stakeholders at multiple levels and can include recommendations on factors that may impact inequities in research partnerships. Existing frameworks such as the Council for International Organizations of Medical Sciences guidelines, UNAIDS-AVAC Good Participatory Practice guidelines, and the WHO/TDR framework for research capacity strengthening have been utilized to support ethical and equitable research practices in global contexts. These frameworks are aligned with foundational ethical declarations, such as the Helsinki Declaration of 1964, which emphasize research equity, non-exploitation, and the protection of human subjects across diverse settings [92]. Grant funders and institutional IRBs can include checklists as an addendum to submitted protocols which may encourage reflexivity and lead to research projects that avoid parachute research, different standards of treatment, unilateral project development or design, and bias.^12^, ^22^, ^34^, ^43^, ^44^, ^70^ Checklists can exist at individual, institutional, and structural levels to account for the unique roles of key stakeholders identified in this review. Other metrics such as decolonization index (D-index) have also been proposed to track global health decolonization in research engagements and can be incorporated into checklist measures.^58^ Establishing measures to quantify the progress of decolonization can be used to evaluate the efficacy of these checklists and of movement in the field. Authorship as a measure has been explored, however further studies looking at change over time, given the increase in literature and discussion about equity in global health collaborations, may be useful to measure progress.^18^, ^21^, ^22^ Tracking changes in the number of journals waiving article processing feeds for LMICs researchers or the introduction of free translation services or non-English publication to mitigate inequities caused by language bias may be useful to access and alleviate larger concerns of publication bias.^12^ Future studies quantitively evaluating changes in funding going towards diseases disproportionally impacting LMICs or capacity building initiatives, leadership in global health governmental and non-governmental organizations, and exploring the perceptions of varying stakeholders on decolonizing research collaborations, are also warranted to measure progress in response to recently established initiatives and increased awareness on the topic.^23^, ^24^, ^47^, ^53^, ^58^, ^70^, ^80^, ^83^, ^91^ A scoping review on decolonizing global health by Amri et al. reported similar findings to this scoping review highlighting consistent themes across the literature and denoting the importance of further research on the topic.

## Limitations

Our study has limitations. The full-text screening of each article for inclusion was not verified by multiple reviewers, increasing the potential risk of bias in included studies. Non-English publications were also excluded. This review was also conducted using two searches at different timepoints which may introduce bias, and the total number of unique articles identified may not be accurate due to overlap in the second search potentially caused by preprint dates. Open AI ChatGBT−4-turbo was used to thematically analyze articles included from the second search potentially limiting bias, as authors who had previously manually identified themes from included articles in the first search may have implicitly recognized past themes to a greater degree. However, Open AI GBT−4-turbo also introduces limitations as there is potential for false information to be produced by artificial intelligence.^92^ For example, incorrect quotes were noticed by authors when Open AI GBT−4-turbo was asked to provide supporting quotes for identified themes during prompt development leading to the exclusion of quotes in the final prompt. Duplicates were also manually identified by individuals screening, not flagged by Covidence, likely due to differences in how the journal name imported and may also affect the total number of unique articles due to potentially missed duplicates. Search strategies and inclusion and exclusion criteria were defined for the original search and resulted in a significant number of potentially eligible articles for second search as literature on the topic increased over time. Due to this, category or thematic saturation was used to determine literature included in this review, and full text screening and conflict resolution was stopped once saturation was reached, however, this technique may result in missing themes. Publication and authorship bias may also play a role in the results of this review as research has reported literature on decolonizing global health predominantly comes from researchers affiliated with HICs.^92^ Future reviews solely including literature with lead authors from LMICs can be conducted to account for this bias.

## Conclusions

Overall, this scoping review implicated the importance of centering local communities and LMIC researchers in global health practice, while highlighting systemic barriers that lead to imbalances in research collaborations between HICs and LMICs. Additionally, it highlighted the importance of social justice and education in creating equitable global health practices. Implementing systems that can help global health move past barriers presented in this review are essential for further research and practice. Findings from this review can translate into global health reform recommendations by contributing to the creation of a checklist modeling decolonized global health practice for stakeholders. By creating a checklist exemplifying specific ways global health can become equitable, participatory, and culturally humble, steps can be made in the right direction.

## CRediT authorship contribution statement

**Canice Christian:** Writing – review & editing, Validation, Project administration, Methodology, Investigation, Formal analysis, Data curation, Conceptualization. **Lucía Abascal Miguel:** Writing – review & editing, Validation, Project administration, Methodology, Investigation, Formal analysis, Data curation, Conceptualization. **Fieldhouse Jane Kees:** Writing – review & editing, Validation, Project administration, Methodology, Investigation, Formal analysis, Data curation, Conceptualization. **Elizabeth Fair:** Writing – review & editing, Validation, Resources, Project administration, Methodology, Investigation, Funding acquisition, Data curation, Conceptualization. **Ali Mirzazadeh:** Writing – review & editing, Writing – original draft, Validation, Supervision, Resources, Project administration, Methodology, Investigation, Funding acquisition, Formal analysis, Data curation, Conceptualization. **Rebekah Kaufman:** Writing – review & editing, Writing – original draft, Visualization, Validation, Software, Project administration, Methodology, Investigation, Formal analysis, Data curation, Conceptualization. **Mariana Garza Zuazua:** Writing – review & editing, Validation, Project administration, Data curation. **Sarah Gallalee:** Writing – review & editing, Project administration, Methodology, Investigation, Data curation, Conceptualization.

## Ethics approval and consent to participate

Human subjects were not involved in this research. All data used is publicly available.

## Consent for publication

Not applicable.

## Funding

Support for this scoping review was provided by an Incubator Award from the University of California Global Health Institute (UCGHI). (PIs: Fair, Mirzazadeh).

## Declaration of Generative AI and AI-assisted technologies in the writing process

We used AI GPT−4-turbo (OpenAI; accessed through ChatGPT Plus in March 2025) as an assistive tool to support thematic identification within the predefined domains of interest. The complete prompt is provided in Appendix Item 5. Details are provided in the Methods section.

## Declaration of Competing Interest

The authors declare that they have no known competing financial interests or personal relationships that could have appeared to influence the work reported in this paper.

## Data Availability

Not applicable.
