# Supplementary material for: Progress and challenges in decolonizing global health research partnerships: A scoping review
Source: Glob Health Res Policy. 2026 Jul 31;11(1):207–17. doi: 10.1016/j.ghrp.2026.07.002 (PMC13734869; doi:10.1016/j.ghrp.2026.07.002)
Supplement: Supplementary file 1 [file mmc1.docx]

**APPENDIX**

**Item 1. Inclusion and Exclusion Criteria**

**Types of study to be included**

All types of peer-review articles (includes commentaries and editorials) discussing global health decolonization and related concepts such as supremacy, and north/south partnership will be included. We include the peer-reviewed literature that entails partnerships/relationships for global health between institutions in low- and middle-income countries (LMICs) that were previously colonized and high-income countries (HICs) that were colonizers. We excluded dissertations, books, reports that may contribute to the topic but are difficult to investigate the overlap / duplicates of their contents with published peer-review articles.

**Condition or domain being studied**

Global health often entails partnerships between institutions in low- and middle-income countries (LMICs) that were previously colonized and high-income countries (HICs) that were colonizers. Little attention has been paid to the legacy of former colonial relationships and the influence they have on global health initiatives. There have been recent calls for the decolonization of global health, and the re-examination of assumptions and practices underpinning global health partnerships. This scoping review is to comprehensively identify and describe global health decolonization, assess the progress made and recognize the existing challenges.

**Participants/population**

Population of interest will be those who work on global health between HICs and LMICs.

**Intervention(s), exposure(s)**

Actions to decolonize global health between HICs and LMICs. We don’t limit the date, but include this as part of the analysis, looking at our results and the dates the articles were published to get a sense of when “decolonizing” or “supremacy” in the context of global health started to be published on as a topic.

**Comparator(s)/control**

None

**Main outcome(s)**

Due to the descriptive characteristics of this scoping review, the outcomes of interest are as follows (reported separately for HICs and LMICs):

1. **Concept and definition** of global health decolonization
2. **Progress** made towards a decolonized global health world (looking at indicators that measures inputs such as initiatives, strategies, operation plan, strategic plans, as well as their output/outcome/impacts.
3. **Barriers** in implementing global health decolonization
4. **Roles** of global health organizations in decolonizing global health

**Item 2. Search Strategies**

PubMed ("Global Health"[Mesh] OR "global health" OR (globali* AND health)) AND (decoloni* OR supremacy OR hegemon*)

Web of Science

("Global Health"[Mesh] OR (global AND health)) AND (decoloni* OR supremacy)

Embase('global health'/exp OR 'global health' OR (('global'/exp OR global) AND ('health'/exp ORhealth))) AND (decolonization OR decolonisation OR decolonizing OR decolonising OR decolonize OR decolonise OR supremacy

Select ProQuest databases

"Global Health" AND (decoloni* OR supremacy)

JSTOR

"Global Health" AND (decoloni* OR supremacy)

**Item 3. Codebook**

| Outcome Code | Description |
| --- | --- |
| Concept | What would decolonized global health look like? Definitions of what decolonization looks like in global health research partnerships |
| Progress | Progress which has been made towards decolonized global health, specific examples of progress, or areas where change has been made. |
| Barriers | Specific barriers to equity in global health. Barriers to decolonized global health practice and partnerships. |
| Roles | The role of specific global health authorities in overcoming barriers to decolonized global health and creating equitable global health partnerships. |

**Item 3. Included Articles**

| **Title** | **Author** | **Type of Paper** | **Topic of Paper** |
| --- | --- | --- | --- |
| Decolonising COVID-19: delaying external debt repayments | Khan 2020 | Commentaries | COVID-19 and debt repayments |
| A time for new north-south relationships in global health | Kim 2017 | Commentaries | Global health relationships |
| Decolonising global health: if not now, when? | Büyüm 2020 | Commentaries | Decolonizing global health and COVID-19 |
| The problem of ‘trickle-down science’ from the Global North to the Global South | Reidpath 2019 | Commentaries | Trickle down science |
| Will global health survive its decolonization? | Abimbola 2020 | Perspectives | Global health decolonization |
| Decolonizing Global Health Education: Rethinking Institutional Partnerships and Approaches | Eichbaum 2021 | Scholarly Perspective | Institutional Partnerships |
| Decolonising COVID-19 | Lancet 2020 | Editorial | COVID-19 |
| Emerging diseases, re-emerging histories | Green 2020 | Spotlight Articles | Infectious Diseases |
| Decolonising global health in the time of COVID-19 | Fofana 2021 | Research Article | COVID-19 and decolonization |
| Global Health -emergence, hegemonic trends and biomedical reductionism | Holst 2020 | Debate | Biomedical Reductionism |
| Rethinking development interventions through the lens of decoloniality in sub-Saharan Africa: The case of global health | Gautier 2020 | Research Article | Development Interventions |
| Decolonising global health: transnational research partnerships under the spotlight | Lawrence 2020 | Research Article | Transnational Research |
| Decolonising research methodologies: lessons from a qualitative research project, Cape Town, South Africa | Keikelame 2019 | Qualitative Research Article | Research Methodologies |
| Indigenous perspectives on education for sustainable healthcare | Redvers 2020 | Research Article | Indigenous Views |
| Decolonising Global (Public) Health: from Western universalism to Global pluriversalities | Affun-Adegbulu 2020 | Commentary | Western Universalism |
| Mutual research capacity strengthening: a qualitative study of two-way partnerships in public health research | Redman-MacLaren 2012 | Research Article | Capacity Building |
| Ethics of Development Assistance for Health | Ruger 2015 | Research Article | Development Assistance for Health |
| New Partnerships for Co-delivery of the 2030 Agenda for Sustainable Development | Bucher 2020 | Research Progress | Partnerships and SDG Goals |
| Enhancing decolonization and knowledge transfer in nursing research with non-western populations: examining the congruence between primary healthcare and postcolonial feminist approaches | Racine 2011 | Feature | Decolonizing nursing research |
| Southern exposure: levelling the Northern tilt in global medical and medical humanities education | Naidu 2020 | Reflections | Northern tilt in medical education |
| COVID-19 opens a window of reflection for comparative health systems and global health research | Agartan 2020 | Perspectives | COVID-19 |
| Stuck in the middle: a systematic review of authorship in collaborative health research in Africa, 2014–2016 | Hedt-Gauthier 2019 | Research Article | Authorship Inequities |
| At the Roots of The World Health Organization's Challenges: Politics and Regionalization | Fee 2016 | Spotlight Article | World Health Organization |
| Owning our madness: Contributions of Jamaican psychiatry to decolonizing Global Mental Health | Hickling 2020 | Article | Global Mental Health |
| Ethical implications of cross-cultural international art therapy | Potash 2017 | Research Article | Ethics of Global Mental Health |
| Racial, Ethnic, Cultural, and National Disparities in Counseling and Psychotherapy Outcome Are Inevitable but Eliminating Global Mental Health Disparities with Indigenous Healing Is Not | Bedi 2018 | Special Selection | Global Mental Health and Indigenous Healing |
| Ethical globalization? Decolonizing theoretical perspectives for internationalization in Canadian medical education | Bhandal 2018 | Research Article | Decolonization in medical education |
| Towards post-colonial capacity-building methodologies - some remarks on the experiences of health researchers from Mozambique and Angola | Carvalho 2019 | Article | Capacity Building |
| Decolonization in health professions education: reflections on teaching through a transgressive pedagogy | Rodney 2016 | Major Contribution | Decolonizing education |
| COVID-19 in sub-Saharan Africa: impacts on vulnerable populations and sustaining home-grown solutions | Wallace 2020 | Special Selection Commentary | COVID-19 and Home-Grown Solutions |
| The coin model of privilege and critical allyship: implications for health | Nixon 2019 | Debate | Critical Allyship |
| Achieving equitable leadership in Global Health partnerships: barriers experienced and strategies to improve grant funding for early- and mid-career researchers. | Chikwari 2024 | Qualitative Inquiry | Local knowledge, capacity strengthening, and funding disparities |
| Barriers to Decolonizing Global Health: Identification of Research Challenges Facing Investigators Residing in Low- and Middle-Income Countries. | Anane-Binfoh 2024 | Qualitative Research Article | Decolonized partnerships and funding structures |
| Decoloniality in global health research: ten tasks for early career researchers. | Keynejad 2023 | Perspective | Decoloniality tasks for early career researchers |
| Applying a Power Analysis to Everything We Do: A Qualitative Inquiry to Decolonize the Global Health and Development Project Cycle. | Tuhebwe 2023 | Qualitative Research Article | Decolonizing the project cycle in global health |
| Making research ethics work for global health: towards a more agile and collaborative approach. | Chattopadhyay 2023 | Policy Analysis | Ethical frameworks for decolonized global health |
| Transforming global health professions education for sustainability. | Naidu 2024 | Education Review | Decolonizing global health education and sustainability |
| A multidimensional account of social justice for global health research. | Pratt 2023 | Conceptual Framework | Social justice and decolonization in global health research |
| Barriers to sustainable health research leadership in the Global South: Time for a Grand Bargain on localization of research leadership? | Abouzeid 2022 | Policy Review | Barriers to sustainable research leadership in the Global South |
| Learner Milestones to Guide Decolonial Global Health Education. | Ratner 2022 | Educational Framework | Milestone-based decolonial training in global health |
| To decolonize global surgery and global health we must be radically intentional. | Foretia 2023 | Commentary | Radical intention in decolonizing surgery and global health |
| A proposed guide to reducing bias and improving assessments of decolonization in global health research | Ngaruiya 2024 | Methodological Commentary | Reducing bias and assessing decolonization |
| A toolkit for decolonizing global emergency medicine education | Dozois 2023 | Perspective | Decolonizing global health education |
| Equitable global health research collaborations with a mind of human dignity | Achana 2023 | Perspective | Human dignity-centered equitable research partnerships |
| ACCESS model: a step toward an empowerment model in global health education | Kalyesubula 2023 | Case Study | ACCESS model for empowerment in global health education |
| Authorship representation in global emergency medicine: a bibliometric analysis from 2016 to 2020 | Garbern 2022 | Bibliometric Analysis | Authorship inequities in global emergency medicine |
| Responsible north-south research and innovation: A framework for transdisciplinary research leadership and management | RISEConsortiumj 2024 | Implementation Study | Transdisciplinary leadership in North-South collaborations |
| An organizing framework to break down Western-centric views of knowledge in North-South research | Turner 2024 | Framework Development | South-North knowledge co-construction |
| Shifting power in global health will require leadership by the Global South and allyship by the Global North | Pai 2024 | Perspective | Global South leadership and North allyship |
| Are we training our students to be white saviours in global health? | Banerjee 2023 | Educational Commentary | White saviorism in global health education |
| Envisioning the futures of global health: three positive disruptions | Sewankambo 2023 | Perspective | Three positive disruptions in global health |
| A long-term process for decolonizing and democratizing community-focused research: the case for MicroResearch in East Africa and in Canada. | MacDonald 2023 | Conceptual Perspective | Futures of global health through dignity and local leadership |
| "We should be at the table together from the beginning": perspectives on partnership from stakeholders at four research institutions in sub-Saharan Africa. | Voller 2022 | Longitudinal Case Study | MicroResearch for democratizing community health research |
| Decolonising global health by decolonising academic publishing. | Khan 2022 | Stakeholder Interview Study | LMIC perspectives on equitable research partnerships |
| Post-decolonisation: Global Health and Global Surgery's Coming of Age. | Chawla 2022 | Publishing Commentary | Decolonizing academic publishing in global health |
| Barriers to decolonising educational partnerships in global health. | Kulesa 2021 | Commentary | Global surgery and equity frameworks |

^*ChatGBT-40 Plus was used to assist in generating this table^

**Item 4. Articles Included by Results Subsection**

| **CONCEPT** | |
| --- | --- |
| Equitable | - Eichbaum QG, Adams LV, Evert J, Ho MJ, Semali IA, van Schalkwyk SC. Decolonizing Global Health Education: Rethinking Institutional Partnerships and Approaches. Acad Med. 2021;96(3):329-35. - Büyüm AM, Kenney C, Koris A, Mkumba L, Raveendran Y. Decolonising global health: if not now, when? BMJ Glob Health. 2020;5(8). - Abimbola S, Pai M. Will global health survive its decolonisation? Lancet. 2020;396(10263):1627-8. - The Lancet Global H. Decolonising COVID-19. The Lancet Global Health. - Fofana MO. Decolonising global health in the time of COVID-19. Glob Public Health. 2021;16(8-9):1155-66. - Redvers N, Schultz C, Vera Prince M, Cunningham M, Jones R, Blondin B. Indigenous perspectives on education for sustainable healthcare. Med Teach. 2020;42(10):1085-90. - Affun-Adegbulu C, Adegbulu O. Decolonising Global (Public) Health: from Western universalism to Global pluriversalities. BMJ Glob Health. 2020;5(8). - Bucher A, Collins A, Heaven Taylor B, Pan D, Visman E, Norris J, et al. New Partnerships for Co-delivery of the 2030 Agenda for Sustainable Development. International Journal of Disaster Risk Science. 2020;11(5):680-5. - Naidu T. Southern exposure: levelling the Northern tilt in global medical and medical humanities education. Adv Health Sci Educ Theory Pract. 2021;26(2):739-52. - Hedt-Gauthier BL, Jeufack HM, Neufeld NH, Alem A, Sauer SM, Odhiambo JA, et al. Stuck in the middle: a systematic review of authorship in collaborative health research in Africa, 2014–2016. BMJ Global Health. 2019;4. - Redman-MacLaren M, MacLaren DJ, Harrington H, Asugeni R, Timothy-Harrington R, Kekeubata E, et al. Mutual research capacity strengthening: a qualitative study of two-way partnerships in public health research. International Journal for Equity in Health. 2012;11(1):79. - Kim JU, Oleribe O, Njie R, Taylor-Robinson SD. A time for new north-south relationships in global health. Int J Gen Med. 2017;10:401-8. - Roxanne CK, Omar D, Rebecca I, et al. Decoloniality in global health research: Ten tasks for early career researchers. BMJ Global Health 2023;8:e014298. - Pratt B. A multidimensional account of social justice for global health research. Bioethics 2023;37:624-636. - Achana F, Boah M, Chirawurah D, et al. Equitable global health research collaborations with a mind of human dignity. Frontiers in Education 2023;Volume 8 – 2023 - Pai M, Bandara S, Kyobutungi C. Shifting power in global health will require leadership by the global south and allyship by the global north. The Lancet 2024;404:1711-1713. - Sewankambo NK, Wallengren E, De Angeles KJC, et al. Envisioning the futures of global health: Three positive disruptions. The Lancet 2023;401:1247-1249. - Banerjee AT, Bandara S, Senga J, et al. Are we training our students to be white saviours in global health? The Lancet 2023;402:520-521. - Naidu T and Ramani S. Transforming global health professions education for sustainability. Medical Education 2024;58:129-135. - Abouzeid M, Muthanna A, Nuwayhid I, et al. Barriers to sustainable health research leadership in the global south: Time for a grand bargain on localization of research leadership? Health Research Policy and Systems 2022;20:136. - Chawla B, Lindert J, Sharma D. Post-decolonisation: Global health and global surgery's coming of age. Indian J Surg 2022;84:259-261. - Kulesa J and Brantuo NA. Barriers to decolonising educational partnerships in global health. BMJ Glob Health 2021;6 |
| Participatory and Power-Sharing | - Eichbaum QG, Adams LV, Evert J, Ho MJ, Semali IA, van Schalkwyk SC. Decolonizing Global Health Education: Rethinking Institutional Partnerships and Approaches. Acad Med. 2021;96(3):329-35. - Abimbola S, Pai M. Will global health survive its decolonisation? Lancet. 2020;396(10263):1627-8. - Redvers N, Schultz C, Vera Prince M, Cunningham M, Jones R, Blondin B. Indigenous perspectives on education for sustainable healthcare. Med Teach. 2020;42(10):1085-90. - Bucher A, Collins A, Heaven Taylor B, Pan D, Visman E, Norris J, et al. New Partnerships for Co-delivery of the 2030 Agenda for Sustainable Development. International Journal of Disaster Risk Science. 2020;11(5):680-5. - Carvalho A, Ferrinho P, Craveiro I. Towards post-colonial capacity-building methodologies - some remarks on the experiences of health researchers from Mozambique and Angola. Cien Saude Colet. 2019;24(5):1617-26. - Keikelame MJ, Swartz L. Decolonising research methodologies: lessons from a qualitative research project, Cape Town, South Africa. Glob Health Action. 2019;12(1):1561175. - Sewankambo NK, Wallengren E, De Angeles KJC, et al. Envisioning the futures of global health: Three positive disruptions. The Lancet 2023;401:1247-1249. - Abouzeid M, Muthanna A, Nuwayhid I, et al. Barriers to sustainable health research leadership in the global south: Time for a grand bargain on localization of research leadership? Health Research Policy and Systems 2022;20:136. - Ratner L, Sridhar S, Rosman SL, et al. Learner milestones to guide decolonial global health  education. Annals of Global Health 2022 - Kalyesubula R, Sadigh M, Okong B, et al. Access model: A step toward an empowerment model in global health education. Frontiers in Education 2023;Volume 8 - 2023 - Banerjee AT, Bandara S, Senga J, et al. Are we training our students to be white saviours in global health? The Lancet 2023;402:520-521. - French MA, Barker SF, Henry R, et al. Responsible north–south research and innovation: A framework for transdisciplinary research leadership and management. Research Policy 2024;53:105048. - MacDonald NE, Bortolussi R, Kabakyenga J. A long-term process for decolonizing and democratizing community-focused research: The case for microresearch in east africa and in canada. Canadian Journal of Public Health 2023;114:147-151. - Khan SA. Decolonising global health by decolonising academic publishing. BMJ Glob Health 2022;7 - Chawla B, Lindert J, Sharma D. Post-decolonisation: Global health and global surgery's coming of age. Indian J Surg 2022;84:259-261.Kulesa J and Brantuo NA. Barriers to decolonising educational partnerships in global health. BMJ Glob Health 2021;6 - Roxanne CK, Omar D, Rebecca I, et al. Decoloniality in global health research: Ten tasks for early career researchers. BMJ Global Health 2023;8:e014298. - Foretia DA. To decolonize global surgery and global health we must be radically intentional. The American Journal of Surgery 2023;225:595-596. - Ngaruiya C, Muhammad MI, Sam-Agudu NA. A proposed guide to reducing bias and improving assessments of decolonization in global health research. Frontiers in Education 2024;Volume 9 – 2024 - Turner H, Rogers B, Kneebone S, et al. An organizing framework to break down western-centric views of knowledge in north-south research. Sustain Sci 2024;19:647-664. - Achana F, Boah M, Chirawurah D, et al. Equitable global health research collaborations with a mind of human dignity. Frontiers in Education 2023;Volume 8 – 2023 - Kulesa J and Brantuo NA. Barriers to decolonising educational partnerships in global health. BMJ Glob Health 2021;6 - Pai M, Bandara S, Kyobutungi C. Shifting power in global health will require leadership by the global south and allyship by the global north. The Lancet 2024;404:1711-1713. - Pratt B. A multidimensional account of social justice for global health research. Bioethics 2023;37:624-636. |
| Promote Cultural Humility and Epistemic Justice | - Redvers N, Schultz C, Vera Prince M, Cunningham M, Jones R, Blondin B. Indigenous perspectives on education for sustainable healthcare. Med Teach. 2020;42(10):1085-90. - Affun-Adegbulu C, Adegbulu O. Decolonising Global (Public) Health: from Western universalism to Global pluriversalities. BMJ Glob Health. 2020;5(8). - Keikelame MJ, Swartz L. Decolonising research methodologies: lessons from a qualitative research project, Cape Town, South Africa. Glob Health Action. 2019;12(1):1561175. - Hickling FW. Owning our madness: Contributions of Jamaican psychiatry to decolonizing Global Mental Health. Transcultural Psychiatry. 2019;57(1):19-31. - Potash JS, Bardot H, Moon CH, Napoli M, Lyonsmith A, Hamilton M. Ethical implications of cross-cultural international art therapy. The Arts in Psychotherapy. 2017;56:74-82. - Bedi RP. Racial, ethnic, cultural, and national disparities in counseling and psychotherapy outcome are inevitable but eliminating global mental health disparities with indigenous healing is not. Archives of Scientific Psychology. 2018;6(1):96-104. - Rodney R. Decolonization in health professions education: reflections on teaching through a transgressive pedagogy. Can Med Educ J. 2016;7(3):e10-e8. - Gautier L, Karambé Y, Dossou JP, Samb OM. Rethinking development interventions through the lens of decoloniality in sub-Saharan Africa: The case of global health. Glob Public Health. 2022;17(2):180-93. - Bhandal T. Ethical globalization? Decolonizing theoretical perspectives for internationalization in Canadian medical education. Can Med Educ J. 2018;9(2):e33-e45. - Naidu T. Southern exposure: levelling the Northern tilt in global medical and medical humanities education. Adv Health Sci Educ Theory Pract. 2021;26(2):739-52. - Redman-MacLaren M, MacLaren DJ, Harrington H, Asugeni R, Timothy-Harrington R, Kekeubata E, et al. Mutual research capacity strengthening: a qualitative study of two-way partnerships in public health research. International Journal for Equity in Health. 2012;11(1):79.c - Naidu T and Ramani S. Transforming global health professions education for sustainability. Medical Education 2024;58:129-135. - Turner H, Rogers B, Kneebone S, et al. An organizing framework to break down western-centric views of knowledge in north-south research. Sustain Sci 2024;19:647-664. - Banerjee AT, Bandara S, Senga J, et al. Are we training our students to be white saviours in global health? The Lancet 2023;402:520-521. - Sewankambo NK, Wallengren E, De Angeles KJC, et al. Envisioning the futures of global health: Three positive disruptions. The Lancet 2023;401:1247-1249. - Khan SA. Decolonising global health by decolonising academic publishing. BMJ Glob Health 2022;7 - Kulesa J and Brantuo NA. Barriers to decolonising educational partnerships in global health. BMJ Glob Health 2021;6 |
| **PROGRESS** | |
| Awareness | - Büyüm AM, Kenney C, Koris A, Mkumba L, Raveendran Y. Decolonising global health: if not now, when? BMJ Glob Health. 2020;5(8). - Fofana MO. Decolonising global health in the time of COVID-19. Glob Public Health. 2021;16(8-9):1155-66. - Naidu T. Southern exposure: levelling the Northern tilt in global medical and medical humanities education. Adv Health Sci Educ Theory Pract. 2021;26(2):739-52. - Gautier L, Karambé Y, Dossou JP, Samb OM. Rethinking development interventions through the lens of decoloniality in sub-Saharan Africa: The case of global health. Glob Public Health. 2022;17(2):180-93. - Lawrence DS, Hirsch LA. Decolonising global health: transnational research partnerships under the spotlight. Int Health. 2020;12(6):518-23. - Chawla B, Lindert J, Sharma D. Post-decolonisation: Global health and global surgery's coming of age. Indian J Surg 2022;84:259-261. - Achana F, Boah M, Chirawurah D, et al. Equitable global health research collaborations with a mind of human dignity. Frontiers in Education 2023;Volume 8 – 2023 - Ratner L, Sridhar S, Rosman SL, et al. Learner milestones to guide decolonial global health  education. Annals of Global Health 2022 - Pratt B. A multidimensional account of social justice for global health research. Bioethics 2023;37:624-636. |
| Leadership | - Reidpath DD, Allotey P. The problem of 'trickle-down science' from the Global North to the Global South. BMJ Glob Health. 2019;4(4):e001719. - Agartan TI. COVID-19 opens a window of reflection for comparative health systems and global health research. New Perspectives on Turkey. 2020;63:190 - 208. - Fee E, Cueto M, Brown TM. At the Roots of The World Health Organization's Challenges: Politics and Regionalization. Am J Public Health. 2016;106(11):1912-7. - Hedt-Gauthier BL, Jeufack HM, Neufeld NH, Alem A, Sauer SM, Odhiambo JA, et al. Stuck in the middle: a systematic review of authorship in collaborative health research in Africa, 2014–2016. BMJ Global Health. 2019;4. - Redman-MacLaren M, MacLaren DJ, Harrington H, Asugeni R, Timothy-Harrington R, Kekeubata E, et al. Mutual research capacity strengthening: a qualitative study of two-way partnerships in public health research. International Journal for Equity in Health. 2012;11(1):79. - Carvalho A, Ferrinho P, Craveiro I. Towards post-colonial capacity-building methodologies - some remarks on the experiences of health researchers from Mozambique and Angola. Cien Saude Colet. 2019;24(5):1617-26. - Ngaruiya C, Muhammad MI, Sam-Agudu NA. A proposed guide to reducing bias and improving assessments of decolonization in global health research. Frontiers in Education 2024;Volume 9 – 2024 |
| Paradigm, Initiatives, and Capacity Building | - Kim JU, Oleribe O, Njie R, Taylor-Robinson SD. A time for new north-south relationships in global health. Int J Gen Med. 2017;10:401-8. - Bhandal T. Ethical globalization? Decolonizing theoretical perspectives for internationalization in Canadian medical education. Can Med Educ J. 2018;9(2):e33-e45. - Lawrence DS, Hirsch LA. Decolonising global health: transnational research partnerships under the spotlight. Int Health. 2020;12(6):518-23. - Kalyesubula R, Sadigh M, Okong B, et al. Access model: A step toward an empowerment model in global health education. Frontiers in Education 2023;Volume 8 – 2023 - Dozois A, González Marqués C, Thilakasiri K, et al. A toolkit for decolonizing global emergency medicine education. Frontiers in Education 2023;Volume 8 – 2023 - French MA, Barker SF, Henry R, et al. Responsible north–south research and innovation: A framework for transdisciplinary research leadership and management. Research Policy 2024;53:105048. - Abouzeid M, Muthanna A, Nuwayhid I, et al. Barriers to sustainable health research leadership in the global south: Time for a grand bargain on localization of research leadership? Health Research Policy and Systems 2022;20:136. - Ngaruiya C, Muhammad MI, Sam-Agudu NA. A proposed guide to reducing bias and improving assessments of decolonization in global health research. Frontiers in Education 2024;Volume 9 – 2024 - Chawla B, Lindert J, Sharma D. Post-decolonisation: Global health and global surgery's coming of age. Indian J Surg 2022;84:259-261. |
| **BARRIERS** | |
| Implicit Bias, Undervaluing, and Knowledge Exclusion | - Büyüm AM, Kenney C, Koris A, Mkumba L, Raveendran Y. Decolonising global health: if not now, when? BMJ Glob Health. 2020;5(8). - Abimbola S, Pai M. Will global health survive its decolonisation? Lancet. 2020;396(10263):1627-8. - Fofana MO. Decolonising global health in the time of COVID-19. Glob Public Health. 2021;16(8-9):1155-66. - Affun-Adegbulu C, Adegbulu O. Decolonising Global (Public) Health: from Western universalism to Global pluriversalities. BMJ Glob Health. 2020;5(8). - Kim JU, Oleribe O, Njie R, Taylor-Robinson SD. A time for new north-south relationships in global health. Int J Gen Med. 2017;10:401-8. - Potash JS, Bardot H, Moon CH, Napoli M, Lyonsmith A, Hamilton M. Ethical implications of cross-cultural international art therapy. The Arts in Psychotherapy. 2017;56:74-82. - Bedi RP. Racial, ethnic, cultural, and national disparities in counseling and psychotherapy outcome are inevitable but eliminating global mental health disparities with indigenous healing is not. Archives of Scientific Psychology. 2018;6(1):96-104. - Gautier L, Karambé Y, Dossou JP, Samb OM. Rethinking development interventions through the lens of decoloniality in sub-Saharan Africa: The case of global health. Glob Public Health. 2022;17(2):180-93. - Reidpath DD, Allotey P. The problem of 'trickle-down science' from the Global North to the Global South. BMJ Glob Health. 2019;4(4):e001719. - Wallace LJ, Nouvet E, Bortolussi R, Arthur JA, Amporfu E, Arthur E, et al. COVID-19 in sub-Saharan Africa: impacts on vulnerable populations and sustaining home-grown solutions. Can J Public Health. 2020;111(5):649-53. - Khan SA. Decolonising global health by decolonising academic publishing. BMJ Glob Health 2022;7 - Tuhebwe D, Brittingham S, Kanagaratnam A, et al. Applying a power analysis to everything we do: A qualitative inquiry to decolonize the global health and development project cycle. Glob Health Sci Pract 2023;11 - Abouzeid M, Muthanna A, Nuwayhid I, et al. Barriers to sustainable health research leadership in the global south: Time for a grand bargain on localization of research leadership? Health Research Policy and Systems 2022;20:136. - Dozois A, González Marqués C, Thilakasiri K, et al. A toolkit for decolonizing global emergency medicine education. Frontiers in Education 2023;Volume 8 – 2023 - Kalyesubula R, Sadigh M, Okong B, et al. Access model: A step toward an empowerment model in global health education. Frontiers in Education 2023;Volume 8 – 2023 - Kulesa J and Brantuo NA. Barriers to decolonising educational partnerships in global health. BMJ Glob Health 2021;6 - Ngaruiya C, Muhammad MI, Sam-Agudu NA. A proposed guide to reducing bias and improving assessments of decolonization in global health research. Frontiers in Education 2024;Volume 9 – 2024 - Turner H, Rogers B, Kneebone S, et al. An organizing framework to break down western-centric views of knowledge in north-south research. Sustain Sci 2024;19:647-664. - Pai M, Bandara S, Kyobutungi C. Shifting power in global health will require leadership by the global south and allyship by the global north. The Lancet 2024;404:1711-1713. |
| Different Standards | - Eichbaum QG, Adams LV, Evert J, Ho MJ, Semali IA, van Schalkwyk SC. Decolonizing Global Health Education: Rethinking Institutional Partnerships and Approaches. Acad Med. 2021;96(3):329-35. - Abimbola S, Pai M. Will global health survive its decolonisation? Lancet. 2020;396(10263):1627-8. - Kim JU, Oleribe O, Njie R, Taylor-Robinson SD. A time for new north-south relationships in global health. Int J Gen Med. 2017;10:401-8. - Gautier L, Karambé Y, Dossou JP, Samb OM. Rethinking development interventions through the lens of decoloniality in sub-Saharan Africa: The case of global health. Glob Public Health. 2022;17(2):180-93. - Bhandal T. Ethical globalization? Decolonizing theoretical perspectives for internationalization in Canadian medical education. Can Med Educ J. 2018;9(2):e33-e45. - Lawrence DS, Hirsch LA. Decolonising global health: transnational research partnerships under the spotlight. Int Health. 2020;12(6):518-23. - Büyüm AM, Kenney C, Koris A, Mkumba L, Raveendran Y. Decolonising global health: if not now, when? BMJ Glob Health. 2020;5(8). - The Lancet Global H. Decolonising COVID-19. The Lancet Global Health. 2020;8(5):e612. - Anane-Binfoh NA, Flaherty KE, Zakariah AN, et al. Barriers to decolonizing global health: Identification of research challenges facing investigators residing in low- and middle-income countries. Global Health: Science and Practice 2024 - Sreeparna C and Bregje de K. Making research ethics work for global health: Towards a more agile and collaborative approach. BMJ Global Health 2023;8:e011415. - Abouzeid M, Muthanna A, Nuwayhid I, et al. Barriers to sustainable health research leadership in the global south: Time for a grand bargain on localization of research leadership? Health Research Policy and Systems 2022;20:136. - Foretia DA. To decolonize global surgery and global health we must be radically intentional. The American Journal of Surgery 2023;225:595-596. - Banerjee AT, Bandara S, Senga J, et al. Are we training our students to be white saviours in global health? The Lancet 2023;402:520-521. |
| Financial and Resource Imbalances | - Bucher A, Collins A, Heaven Taylor B, Pan D, Visman E, Norris J, et al. New Partnerships for Co-delivery of the 2030 Agenda for Sustainable Development. International Journal of Disaster Risk Science. 2020;11(5):680-5. - Lawrence DS, Hirsch LA. Decolonising global health: transnational research partnerships under the spotlight. Int Health. 2020;12(6):518-23. - Khan M, Shanks S. Decolonising COVID-19: delaying external debt repayments. The Lancet Global Health. 2020;8(7):e897. - Holst J. Global Health - emergence, hegemonic trends and biomedical reductionism. Global Health. 2020;16(1):42. - Ruger JP. Ethics of Development Assistance for Health. Hastings Center Report. 2015;45(3):23-6. - Anane-Binfoh NA, Flaherty KE, Zakariah AN, et al. Barriers to decolonizing global health: Identification of research challenges facing investigators residing in low- and middle-income countries. Global Health: Science and Practice 2024 - Abouzeid M, Muthanna A, Nuwayhid I, et al. Barriers to sustainable health research leadership in the global south: Time for a grand bargain on localization of research leadership? Health Research Policy and Systems 2022;20:136. - Kalyesubula R, Sadigh M, Okong B, et al. Access model: A step toward an empowerment model in global health education. Frontiers in Education 2023;Volume 8 – 2023 - Pai M, Bandara S, Kyobutungi C. Shifting power in global health will require leadership by the global south and allyship by the global north. The Lancet 2024;404:1711-1713. - MacDonald NE, Bortolussi R, Kabakyenga J. A long-term process for decolonizing and democratizing community-focused research: The case for microresearch in east africa and in canada. Canadian Journal of Public Health 2023;114:147-151. - Achana F, Boah M, Chirawurah D, et al. Equitable global health research collaborations with a mind of human dignity. Frontiers in Education 2023;Volume 8 - 2023 |
| Power and Leadership Imbalances | - Büyüm AM, Kenney C, Koris A, Mkumba L, Raveendran Y. Decolonising global health: if not now, when? BMJ Glob Health. 2020;5(8). - Abimbola S, Pai M. Will global health survive its decolonisation? Lancet. 2020;396(10263):1627-8. - Hedt-Gauthier BL, Jeufack HM, Neufeld NH, Alem A, Sauer SM, Odhiambo JA, et al. Stuck in the middle: a systematic review of authorship in collaborative health research in Africa, 2014–2016. BMJ Global Health. 2019;4. - Kim JU, Oleribe O, Njie R, Taylor-Robinson SD. A time for new north-south relationships in global health. Int J Gen Med. 2017;10:401-8. - Khan M, Shanks S. Decolonising COVID-19: delaying external debt repayments. The Lancet Global Health. 2020;8(7):e897. - Holst J. Global Health - emergence, hegemonic trends and biomedical reductionism. Global Health. 2020;16(1):42. - MacDonald NE, Bortolussi R, Kabakyenga J. A long-term process for decolonizing and democratizing community-focused research: The case for microresearch in east africa and in canada. Canadian Journal of Public Health 2023;114:147-151. - Garbern SC, Hyuha G, González Marqués C, et al. Authorship representation in global emergency medicine: A bibliometric analysis from 2016 to 2020. BMJ Glob Health 2022;7 - Abouzeid M, Muthanna A, Nuwayhid I, et al. Barriers to sustainable health research leadership in the global south: Time for a grand bargain on localization of research leadership? Health Research Policy and Systems 2022;20:136. - Foretia DA. To decolonize global surgery and global health we must be radically intentional. The American Journal of Surgery 2023;225:595-596. - Ngaruiya C, Muhammad MI, Sam-Agudu NA. A proposed guide to reducing bias and improving assessments of decolonization in global health research. Frontiers in Education 2024;Volume 9 – 2024 - Achana F, Boah M, Chirawurah D, et al. Equitable global health research collaborations with a mind of human dignity. Frontiers in Education 2023;Volume 8 – 2023 - French MA, Barker SF, Henry R, et al. Responsible north–south research and innovation: A framework for transdisciplinary research leadership and management. Research Policy 2024;53:105048. - Anane-Binfoh NA, Flaherty KE, Zakariah AN, et al. Barriers to decolonizing global health: Identification of research challenges facing investigators residing in low- and middle-income countries. Global Health: Science and Practice 2024 - Khan SA. Decolonising global health by decolonising academic publishing. BMJ Glob Health 2022;7 - Turner H, Rogers B, Kneebone S, et al. An organizing framework to break down western-centric views of knowledge in north-south research. Sustain Sci 2024;19:647-664. |
| **ROLES** | |
| Individual Researchers | - Eichbaum QG, Adams LV, Evert J, Ho MJ, Semali IA, van Schalkwyk SC. Decolonizing Global Health Education: Rethinking Institutional Partnerships and Approaches. Acad Med. 2021;96(3):329-35. - Abimbola S, Pai M. Will global health survive its decolonisation? Lancet. 2020;396(10263):1627-8. - Affun-Adegbulu C, Adegbulu O. Decolonising Global (Public) Health: from Western universalism to Global pluriversalities. BMJ Glob Health. 2020;5(8). - Potash JS, Bardot H, Moon CH, Napoli M, Lyonsmith A, Hamilton M. Ethical implications of cross-cultural international art therapy. The Arts in Psychotherapy. 2017;56:74-82. - Racine L, Petrucka P. Enhancing decolonization and knowledge transfer in nursing research with non-western populations: examining the congruence between primary healthcare and postcolonial feminist approaches. Nursing Inquiry. 2011;18(1):12-20. - Nixon SA. The coin model of privilege and critical allyship: implications for health. BMC Public Health. 2019;19(1):1637. - Büyüm AM, Kenney C, Koris A, Mkumba L, Raveendran Y. Decolonising global health: if not now, when? BMJ Glob Health. 2020;5(8). - Banerjee AT, Bandara S, Senga J, et al. Are we training our students to be white saviours in global health? The Lancet 2023;402:520-521. - Ratner L, Sridhar S, Rosman SL, et al. Learner milestones to guide decolonial global health  education. Annals of Global Health 2022 - Kalyesubula R, Sadigh M, Okong B, et al. Access model: A step toward an empowerment model in global health education. Frontiers in Education 2023;Volume 8 – 2023 - Turner H, Rogers B, Kneebone S, et al. An organizing framework to break down western-centric views of knowledge in north-south research. Sustain Sci 2024;19:647-664. - Sewankambo NK, Wallengren E, De Angeles KJC, et al. Envisioning the futures of global health: Three positive disruptions. The Lancet 2023;401:1247-1249. - Chikwari CD, Tadesse AW, Shanaube K, et al. Achieving equitable leadership in global health partnerships: Barriers experienced and strategies to improve grant funding for early- and mid-career researchers. BMC Global and Public Health 2024;2:17. - Roxanne CK, Omar D, Rebecca I, et al. Decoloniality in global health research: Ten tasks for early career researchers. BMJ Global Health 2023;8:e014298. - Kulesa J and Brantuo NA. Barriers to decolonising educational partnerships in global health. BMJ Glob Health 2021;6 |
| Academic Institutions | - Naidu T. Southern exposure: levelling the Northern tilt in global medical and medical humanities education. Adv Health Sci Educ Theory Pract. 2021;26(2):739-52. - Carvalho A, Ferrinho P, Craveiro I. Towards post-colonial capacity-building methodologies - some remarks on the experiences of health researchers from Mozambique and Angola. Cien Saude Colet. 2019;24(5):1617-26. - Keikelame MJ, Swartz L. Decolonising research methodologies: lessons from a qualitative research project, Cape Town, South Africa. Glob Health Action. 2019;12(1):1561175. - Lawrence DS, Hirsch LA. Decolonising global health: transnational research partnerships under the spotlight. Int Health. 2020;12(6):518-23. - Naidu T and Ramani S. Transforming global health professions education for sustainability. Medical Education 2024;58:129-135. - Sreeparna C and Bregje de K. Making research ethics work for global health: Towards a more agile and collaborative approach. BMJ Global Health 2023;8:e011415. - Banerjee AT, Bandara S, Senga J, et al. Are we training our students to be white saviours in global health? The Lancet 2023;402:520-521. - Pratt B. A multidimensional account of social justice for global health research. Bioethics 2023;37:624-636. - Ratner L, Sridhar S, Rosman SL, et al. Learner milestones to guide decolonial global health  education. Annals of Global Health 2022 - Ngaruiya C, Muhammad MI, Sam-Agudu NA. A proposed guide to reducing bias and improving assessments of decolonization in global health research. Frontiers in Education 2024;Volume 9 – 2024 - Achana F, Boah M, Chirawurah D, et al. Equitable global health research collaborations with a mind of human dignity. Frontiers in Education 2023;Volume 8 – 2023 - Kulesa J and Brantuo NA. Barriers to decolonising educational partnerships in global health. BMJ Glob Health 2021;6 - Chikwari CD, Tadesse AW, Shanaube K, et al. Achieving equitable leadership in global health partnerships: Barriers experienced and strategies to improve grant funding for early- and mid-career researchers. BMC Global and Public Health 2024;2:17. - Kalyesubula R, Sadigh M, Okong B, et al. Access model: A step toward an empowerment model in global health education. Frontiers in Education 2023;Volume 8 – 2023 - Turner H, Rogers B, Kneebone S, et al. An organizing framework to break down western-centric views of knowledge in north-south research. Sustain Sci 2024;19:647-664. - Pai M, Bandara S, Kyobutungi C. Shifting power in global health will require leadership by the global south and allyship by the global north. The Lancet 2024;404:1711-1713. - French MA, Barker SF, Henry R, et al. Responsible north–south research and innovation: A framework for transdisciplinary research leadership and management. Research Policy 2024;53:105048. |
| Structural Level Stakeholders: Governments, Global Health Organizations, Academic Journals, and Funders | - Abimbola S, Pai M. Will global health survive its decolonisation? Lancet. 2020;396(10263):1627-8. - Bucher A, Collins A, Heaven Taylor B, Pan D, Visman E, Norris J, et al. New Partnerships for Co-delivery of the 2030 Agenda for Sustainable Development. International Journal of Disaster Risk Science. 2020;11(5):680-5. - Wallace LJ, Nouvet E, Bortolussi R, Arthur JA, Amporfu E, Arthur E, et al. COVID-19 in sub-Saharan Africa: impacts on vulnerable populations and sustaining home-grown solutions. Can J Public Health. 2020;111(5):649-53. - French MA, Barker SF, Henry R, et al. Responsible north–south research and innovation: A framework for transdisciplinary research leadership and management. Research Policy 2024;53:105048. - MacDonald NE, Bortolussi R, Kabakyenga J. A long-term process for decolonizing and democratizing community-focused research: The case for microresearch in east africa and in canada. Canadian Journal of Public Health 2023;114:147-151. - Kalyesubula R, Sadigh M, Okong B, et al. Access model: A step toward an empowerment model in global health education. Frontiers in Education 2023;Volume 8 – 2023 - Foretia DA. To decolonize global surgery and global health we must be radically intentional. The American Journal of Surgery 2023;225:595-596. - Abouzeid M, Muthanna A, Nuwayhid I, et al. Barriers to sustainable health research leadership in the global south: Time for a grand bargain on localization of research leadership? Health Research Policy and Systems 2022;20:136. - Chikwari CD, Tadesse AW, Shanaube K, et al. Achieving equitable leadership in global health partnerships: Barriers experienced and strategies to improve grant funding for early- and mid-career researchers. BMC Global and Public Health 2024;2:17. - Dozois A, González Marqués C, Thilakasiri K, et al. A toolkit for decolonizing global emergency medicine education. Frontiers in Education 2023;Volume 8 – 2023 - Ngaruiya C, Muhammad MI, Sam-Agudu NA. A proposed guide to reducing bias and improving assessments of decolonization in global health research. Frontiers in Education 2024;Volume 9 – 2024 - Achana F, Boah M, Chirawurah D, et al. Equitable global health research collaborations with a mind of human dignity. Frontiers in Education 2023;Volume 8 - 2023 - Garbern SC, Hyuha G, González Marqués C, et al. Authorship representation in global emergency medicine: A bibliometric analysis from 2016 to 2020. BMJ Glob Health 2022;7 - Pai M, Bandara S, Kyobutungi C. Shifting power in global health will require leadership by the global south and allyship by the global north. The Lancet 2024;404:1711-1713. - Sewankambo NK, Wallengren E, De Angeles KJC, et al. Envisioning the futures of global health: Three positive disruptions. The Lancet 2023;401:1247-1249. - Khan SA. Decolonising global health by decolonising academic publishing. BMJ Glob Health 2022;7 |

**Item 5. ChatGBT-40 Plus Prompt**

Please summarize the attached article. Only use the information discussed in the article's result section. If the article is a commentary or perspective piece, focus on the analytical core/results like content. If an outcome of interest is not discussed, do not identify anything. Do not provide quotes.

Outcomes of interest:

Concept and Definition: What would decolonized global health look like? Definitions of what decolonization looks like in global health research partnerships

Progress: Progress which has been made towards decolonized global health, specific examples of progress, or areas where change has been made in collaborative research.

Barriers: Specific barriers to equity in global health. Barriers to decolonized global health practice and partnerships.

Roles: The role of specific global health authorities in overcoming barriers to decolonized global health and creating equitable global health partnerships.
